# Deep Learning-Based Inference of Mutational Signature Exposures from Histology Across Tumour Types

**DOI:** 10.3390/cancers18213446

**Published:** 2026-10-25

**Authors:** Byeong Chang Sim, JunSik Kim, Jiyeon Park, YunJi Kim, JungHyun Park, Taehoon Ko, Ji-Won Chun, Tae-Min Kim

**Affiliations:** 1 CMC Institute for Basic Medical Science, The Catholic Medical Center, The Catholic University of Korea, Seoul 06591, Republic of Korea; 202553049@catholic.ac.kr (B.C.S.); 22501836@cmcnu.or.kr (J.K.); pjy0088@catholic.ac.kr (J.P.); yunjikim@cmcnu.or.kr (Y.K.); 22602175@cmcnu.or.kr (J.P.); thko@catholic.ac.kr (T.K.); jwchun@catholic.ac.kr (J.-W.C.); 2 Department of Medical Informatics, College of Medicine, The Catholic University of Korea, Seoul 06591, Republic of Korea; 3 Graduate School, The Catholic University of Korea, Seoul 06591, Republic of Korea; 4 Cancer Research Institute, College of Medicine, The Catholic University of Korea, Seoul 06591, Republic of Korea

**Keywords:** computational pathology, whole-slide image, mutational signature, single-base substitution, multiple-instance learning, genomic instability

## Abstract

Background/Objectives: Mutational signatures reflect the processes that shape cancer genomes. We investigated whether continuous mutational-signature exposures could be inferred from routine histology across tumour types. Methods: We developed whole-slide image regression models for 30 single-base substitution (SBS) signatures in 2741 The Cancer Genome Atlas (TCGA) patients across seven tumour types and evaluated them in 580 Clinical Proteomic Tumor Analysis Consortium (CPTAC) patients. RetCCL mean-feature ridge regression and RetCCL/CTransPath attention-based multiple-instance learning were combined by fixed unweighted averaging. Final ensemble outputs were standardised SBS scores. Patient-disjoint evaluation, full-panel false discovery rate (FDR) adjustment and tumour-labelled slide sensitivity were performed. Results: Among 185 pairs with correlations available in both cohorts, 73 showed positive correlations in both cohorts. Fourteen had nominal positive-direction *p* < 0.05 in both, including breast cancer–SBS3 (TCGA r = 0.289, CPTAC r = 0.215) and lung adenocarcinoma–SBS4 (r = 0.128 and 0.263). Cohort-specific FDR-adjusted values are provided for all 210 pairs. All 14 associations remained positive after restricting inputs to tumour-labelled slides. The SBS3 score correlated with continuous homologous recombination deficiency (r = 0.332). Exploratory TCGA SBS3, SBS6 and SBS10 scores yielded areas under the receiver operating characteristic curve of 0.693, 0.619 and 0.628 for homologous recombination deficiency-high, microsatellite instability and qualifying *POLE*/*POLD1* mutations. External MSI and polymerase AUCs were 0.573 and 0.680, respectively. Conclusions: Histology-derived scores were associated with selected genomic SBS exposures in both cohorts. Further studies are needed to identify the tissue features underlying these associations and assess their clinical relevance.

## Simple Summary

Mutational signatures record the processes that shape cancer genomes, including DNA repair defects and exposure to mutagens. We investigated whether routine pathology images could infer the relative activity of these signatures across seven cancer types. Models were developed using 2741 patients and evaluated in a separate cohort of 580 patients. Fourteen cancer–signature combinations showed nominal positive associations in both cohorts. Multiple-comparison-adjusted values are also reported for the complete panel. Additional analyses examined how the choice of slides affected the results. We also assessed links between image-derived scores and genomic features related to DNA repair and instability. The models produced relative scores associated with specific mutational signatures in both cohorts. Further studies are needed to interpret the tissue features underlying these associations and assess their clinical value.

## 1. Introduction

Cancer genomes accumulate somatic mutations through diverse endogenous and exogenous mutational processes, including spontaneous DNA damage, replication errors, defective DNA repair, and exposure to environmental mutagens. These processes leave characteristic sequence patterns in cancer genomes, serving as molecular fossils of genomic processes that have operated throughout tumour evolution. Computational deconvolution of these sequence context patterns has resolved them into a repertoire of mutational signatures [1]. For example, the single-nucleotide variant catalogue of a tumour can be decomposed into single-base substitution (SBS) signatures, with the exposure of each signature quantifying its contribution to the overall mutational catalogue. A number of SBS signatures have been linked to specific biological processes, e.g., SBS1 is a clock-like, age-associated signature. SBS2 and SBS13 reflect APOBEC cytidine deaminase activity. SBS3 is associated with homologous recombination deficiency (HRD) [2]. SBS6 is associated with mismatch repair (MMR) deficiency and microsatellite instability (MSI) [3]. Unlike individual driver mutations, mutational signatures integrate the cumulative consequences of endogenous and exogenous processes that have shaped a tumour genome.

Histopathological examination of surgical resections and tissue biopsies has long been central to cancer diagnosis and clinical evaluation. Computational pathology has further shown that routine haematoxylin and eosin (H&E) morphology contains information about driver mutations, molecular subtypes, gene expression programmes, MSI, HRD and other genomic phenotypes [4,5,6,7,8]. More focused studies have directly predicted MSI or HRD from whole-slide images (WSIs), including pan-cancer and externally validated settings [5,9,10,11,12]. In these studies, the molecular phenotype itself is generally used as the image-level prediction target. Mutational signatures have also been used as genomic readouts of upstream repair defects. Valieris et al. used Signature 3 and Signature 20 activities to define HRD and MMR-deficient labels for histology-based classification rather than attempting to predict the continuous signature activities themselves [13]. Together, these studies establish links between histology and clinically interpretable consequences of mutational processes. They provide a basis for asking whether histology also contains information about the quantitative activities of the underlying signatures.

A preprint describing the Fuji multimodal pathology foundation model has included binary mutational-signature inference among its training tasks [14], providing a further example of molecular supervision based on mutational signatures. However, systematic prediction of continuous SBS exposures across multiple tumour types, with evaluation of the same tumour-type signature targets in an independent cohort, remains largely uncharacterised. This task is challenging because SBS exposures are continuous, frequently zero-inflated, and strongly dependent on cancer type. Attention-based multiple-instance learning (MIL) enables weakly supervised WSI analysis by aggregating information across thousands of tiles without requiring tile-level annotations [15]. Encoders pretrained on histopathology images can transform individual tiles into informative morphological representations [16,17], while regression-based WSI approaches have established the feasibility of predicting continuous molecular phenotypes [12]. We therefore evaluated whether continuous SBS1–SBS30 exposures could be inferred from tumour histology across seven tumour types, using cancer-specific models trained in TCGA and evaluated in tumour-type-matched CPTAC cohorts. Continuous exposures were the regression targets. The ensemble outputs were standardised SBS scores evaluated against those targets. In exploratory analyses, we additionally asked whether histology-derived SBS3, SBS6 and SBS10 scores retained information about their biologically related genomic features, namely, HRD, MSI, and *POLE*/*POLD1* proofreading-domain mutations, without training the image models directly on those downstream labels.

## 2. Materials and Methods

### 2.1. Study Cohorts

H&E whole-slide images and genomic data were assembled from TCGA and the NCI Genomic Data Commons for breast invasive carcinoma (BRCA), head and neck squamous cell carcinoma (HNSC), kidney renal clear cell carcinoma (KIRC), lung adenocarcinoma (LUAD), lung squamous cell carcinoma (LUSC), ovarian serous cystadenocarcinoma (OV), and uterine corpus endometrial carcinoma (UCEC) [18,19]. Tumour-type-matched CPTAC cohorts were obtained from the CPTAC resource, including the endometrial carcinoma study [20,21]. Analysis-specific sample sizes varied according to the availability of genomic targets and model predictions. WSI and genomic data were matched by patient identifier within each cohort. Patient characteristics, specimen annotations and the patient selection flow are summarised in Supplementary Data S1.

### 2.2. Mutation Signature Evaluation

Exome-level somatic mutation data were used for TCGA and CPTAC. Single-nucleotide variants were used to derive SBS signatures. Proportional SBS1–SBS30 exposures were estimated with deconstructSigs [22] using the built-in signatures.cosmic legacy reference, default trinucleotide normalisation (tri.counts.method = “default”), more than 50 observed substitutions, and a signature cutoff of 0.06. Default normalisation and a 0.06 contribution cutoff have been used with COSMIC 30-signature references in a previous whole-exome sequencing study [23]. deconstructSigs was applied separately to each eligible genomic tumour sample. When multiple eligible genomic samples mapped to the same patient, proportional weights were averaged to obtain a patient-level target. Exposure proportions supplied the molecular training targets. Final image-derived outputs were standardised scores. Target distributions, zero fractions and cohort-level substitution count and eligibility summaries are provided in Supplementary Data S3. Genomic samples and patients are reported separately. To assess dependence on target estimation, the same image-derived scores were compared with saved Sigfit (v1.2.0) legacy-SBS1–SBS30 estimates and SigProfilerAssignment estimates using COSMIC v2, v3.5 and v3.6 (software v1.1.5 for COSMIC v2 and v3.6; v1.1.4 for COSMIC v3.5). Each original-versus-alternative comparison used the same matched patients. Only matching signature names were compared. Split signatures were not combined. Source-specific sample coverage and comparison results are provided in Supplementary Data S3 (signature_comparison).

### 2.3. Image Processing and Feature Extraction

Slides were processed with the End2End and Marugoto regression workflow [12]. WSIs were resampled to 1.143 μm per pixel, and non-overlapping 224 × 224-pixel tiles, representing 256 × 256 μm, were extracted after Canny-edge tissue filtering [24]. Stain appearance was normalised with the Macenko method [25]. Frozen RetCCL ResNet-50 (2048-dimensional) and CTransPath (768-dimensional) features [16,17] were coordinate-aligned. Separate learned 128-dimensional branches were concatenated and fused into a 256-dimensional tile representation. For patients with multiple eligible slides, aligned tiles from all slides were concatenated before bag sampling with a fixed patient-specific seed. The standardised analysis used patient-level RetCCL mean features and multi-output gated-attention MIL bags of 1024 and 2048 tiles. Encoder weights were not updated during downstream model fitting. Tile quality control (QC) scores combined occupancy, tissue fraction, edge density and focus. The formula, thresholds, component definitions and fixed averaging rule are provided in Supplementary Data S1. Software details and recorded environment versions are provided in Supplementary Data S1 and the public code repository.

Image inputs included all matched eligible slides. No pixel-level tumour-region mask or Tumor_Segment_Acceptable restriction was applied. TCGA inputs comprised 5135 primary-tumour-labelled slides (1800 diagnostic FFPE and 3335 frozen tissue sections). CPTAC inputs comprised 2239 slides (1509 tumour-labelled, 638 normal-labelled and 92 with unspecified tissue). Embedding was recorded as FFPE for 1571 slides, OCT for 262 and unavailable in the matched extract for 406. Among 580 CPTAC patients, 451 had at least one normal-labelled slide, and 10 had no tumour-labelled slide. Labels describe the submitted slide, not the tumour fraction of every tile. Cohort-specific slide type and specimen preparation summaries are provided in Supplementary Data S3.

### 2.4. WSI-Based SBS Prediction

The analysis evaluated all 210 combinations of seven tumour types and SBS1–SBS30. A separate cancer-specific model jointly predicted all 30 proportional exposures. TCGA evaluation used patient-disjoint shuffled five-fold cross-validation with three independent repetitions. Within each outer-training partition, 20% of patients were reserved for model selection. At each 1024- and 2048-tile scale, we compared random sampling without replacement, with equal selection probability for each tile, against a quality hybrid scheme. In the latter, half of each bag comprised the tiles with the highest QC scores. The remaining half was sampled at random without replacement from the other tiles. Both schemes were evaluated with or without fusion of the target attention representation with the mean and standard deviation of encoded tiles within the sampled bag. The selected variant in each scale, cancer, seed and fold maximised the stored inner-validation median Pearson correlation and produced all 30 outputs in that context. MIL optimisation used AdamW (learning rate 10^−4^, weight decay 10^−4^, gradient-norm clipping 5.0), at most 25 epochs and patience 8. The loss was Hellinger composition loss plus 0.25 times weighted signature-presence binary cross-entropy, 0.5 times square-root exposure mean-squared error and 0.10 times macro Pearson loss. Checkpoint scoring included SBS outputs with at least three non-zero inner-validation targets and non-zero target variance. The principal component analysis (PCA)-ridge component used RetCCL patient mean features from the inner-training patients, training-fitted standardisation, 16 principal components and ridge alpha 100. It was fitted to square-root exposures, with non-negative predictions squared back to the exposure scale. The PCA-ridge, 1024-tile MIL and 2048-tile MIL outputs were each standardised using the corresponding inner-validation mean and population standard deviation, then combined by a fixed unweighted 1:1:1 arithmetic mean. The resulting outputs were standardised SBS scores, not calibrated exposure proportions. TCGA out-of-fold scores were averaged across the three repetition-specific predictions for each patient. CPTAC predictions were averaged across the 15 TCGA-trained models. Model specifications are in Supplementary Data S1. Full performance results are in Supplementary Data S3.

### 2.5. Exploratory Associations of SBS Scores with Genomic Features

Exploratory genomic-feature associations used TCGA out-of-fold primary SBS3, SBS6 and SBS10 scores for BRCA HRD, UCEC MSI and UCEC polymerase status, respectively. All patients with the corresponding SBS score and required clinical label were included. Genomic-feature labels were used only for evaluation and were not used to fit, select or recalibrate the signature scores. The continuous HRD score was the sum of loss of heterozygosity, telomeric allelic imbalance and large-scale state transition scores [26], with HRD-high defined as ≥42 [27]. The UCEC MSI status was defined using MANTIS [28] scores from the published TCGA pan-cancer analysis [29]. For the present study, scores > 0.6 were classified as positive and scores < 0.4 as negative. Scores from 0.4 to 0.6, inclusive, and missing values were excluded. This yielded 365 of the 422 primary UCEC patients. The originally specified polymerase endpoint was a composite. A patient was positive if at least one of the exact literature-informed qualifying proofreading-domain missense substitutions in *POLE* OR *POLD1* listed in Supplementary Data S2 was identified, and negative if no listed substitution was identified. Double-positive patients were counted once. Negative status denotes absence of a qualifying recorded substitution rather than complete *POLE*/*POLD1* wild type. The 11 *POLE* substitutions match the somatic endometrial carcinoma list in León-Castillo et al., Table 3 [30]. The seven *POLD1* substitutions are a study-defined selection supported by the functional, somatic signature and familial cancer literature [3,31,32]. This research selection is distinguished from germline clinical pathogenicity classification.

External analyses assessed the primary CPTAC UCEC SBS6 and SBS10 scores against source-published MSI-H versus MSS status and the original *POLE* OR *POLD1* composite, respectively. The same variant list and patient-level OR rule in Supplementary Data S2 were applied to the source-publication mutation event annotations (cBioPortal ucec_cptac_2020_mutations, queried using the complete 95-sample ucec_cptac_2020_sequenced list [21]). All 82 evaluated patients were present in that sequenced sample list. Compatible CPTAC BRCA genomic-scar HRD scores or HRD-high labels (score ≥ 42) were not obtained from the CPTAC sources examined. MANTIS sensitivity compared the original TCGA < 0.4/>0.6 rule with a >0.4-positive/≤0.4-negative rule using all available continuous scores [28]. This changes the classification rule and evaluable patient set together, while retaining the image scores.

### 2.6. Statistical Analysis

Pearson correlation between each standardised image-derived score and its observed exposure was the primary association measure, with patient-level Fisher-z 95% confidence intervals. Positive-direction tests (H1: ρ > 0) were retained for all 210 tumour type–signature pairs. Benjamini–Hochberg adjustment was applied separately to all 210 tests in each cohort [33]. An FDR threshold of 0.20 was used for this exploratory analysis to reduce the risk of overlooking potentially relevant associations. The original nominal *p* < 0.05 criterion in both cohorts was retained to identify recurring positive associations. Full effects, confidence intervals and raw/adjusted *p*-values are provided in Supplementary Data S3. Positive–positive, negative–negative and discordant pairs were reported separately. Spearman correlations used the same predictions. Sparse targets were interpreted with their distributions and non-zero counts.

Genomic-feature AUCs were calculated directly from the specified signature scores. AUC confidence intervals used 10,000 class-stratified patient-bootstrap replicates and percentile limits. Continuous SBS3–HRD associations used two-sided Pearson tests, and binary comparisons used two-sided Mann–Whitney U tests. Unadjusted two-sided *p*-values are reported for these exploratory associations.

### 2.7. Image-Input Sensitivity Analysis

All 210 combinations of seven CPTAC cancer types and 30 SBS targets were assessed using tile features selected by source-slide annotation. Tile embeddings from normal-labelled and unspecified source slides were excluded, and those from explicitly tumour-labelled source slides were retained. Retained tile features were pooled per patient and sampled into the original MIL bags. RetCCL patient-mean features for PCA–ridge were also recomputed from retained tiles. The genomic targets and component averaging were retained. Across the seven cancer types, both input conditions used the same 570 retained patients, with 2221 all-input slides versus 1509 tumour-labelled slides. Ten patients without a tumour-labelled input were excluded only from this supplementary assessment. The original 580-patient assessment remained primary. Complete paired correlations, confidence intervals, raw *p*-values and restricted-input cohort-adjusted values are supplied in Supplementary Data S3. Figure S3 displays the original 14 nominal examples. Specimen groups and normal-slide inclusion were compared descriptively using existing all-slide scores, with patient counts reported. Source-slide annotations do not identify the tumour at the tile level.

### 2.8. Attention Visualisation

For each of UCEC–SBS13, UCEC–SBS3, BRCA–SBS3 and LUAD–SBS4 in TCGA and CPTAC, an illustrative patient was selected using the median positive genomic exposure among patients with a tumour-labelled slide. Original MIL weights were displayed at sampled coordinates. The H&E overview, overlay and higher-/lower-weight tiles show how one MIL component weights the sampled regions during aggregation. Each display reports the patient genomic exposure and standardised ensemble score. Colour indicates the within-patient attention rank, and unselected regions are uncoloured. Expert pathological interpretation was not performed.

## 3. Results

### 3.1. Study Population and Analysis Framework

**Figure 1:**
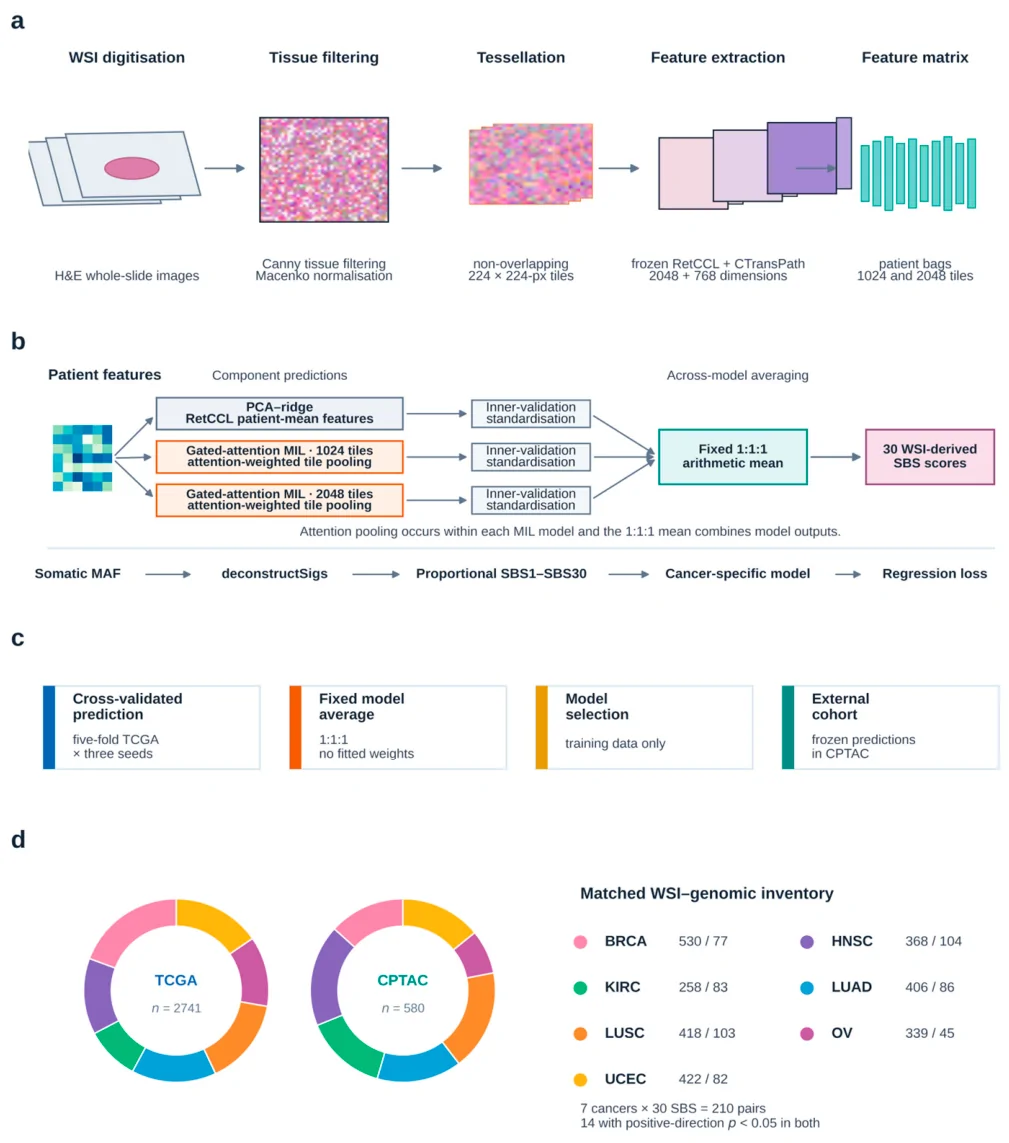
Study cohorts and workflow for histology-based SBS analysis. (**a**) H&E whole-slide images underwent Canny-based tissue filtering, Macenko stain normalisation and non-overlapping 224 × 224-pixel tessellation at 1.143 μm per pixel before frozen RetCCL and CTransPath feature extraction. (**b**) For the 210-pair analysis, each gated-attention MIL component formed a patient representation by attention-weighted tile aggregation. Its SBS outputs and the PCA ridge outputs were standardised using inner-validation values and combined by a fixed unweighted 1:1:1 arithmetic mean. (**c**) Patient-disjoint TCGA cross-validation, model selection within the training data, fixed unweighted model averaging and CPTAC prediction. Primary TCGA out-of-fold SBS3, SBS6 and SBS10 scores were assessed against corresponding genomic features. (**d**) Matched WSI–genomic patient counts by cancer and cohort. Arrows indicate the direction of processing and prediction. Colours distinguish workflow components in panels (**a**–**c**) and cancer types in panel d, as indicated by the labels and legend. Abbreviations: single-base substitution (SBS), haematoxylin and eosin (H&E), whole-slide image (WSI), multiple-instance learning (MIL), principal component analysis (PCA), quality control (QC), The Cancer Genome Atlas (TCGA), Clinical Proteomic Tumor Analysis Consortium (CPTAC), homologous recombination deficiency (HRD), microsatellite instability (MSI), breast invasive carcinoma (BRCA), head and neck squamous cell carcinoma (HNSC), kidney renal clear cell carcinoma (KIRC), lung adenocarcinoma (LUAD), lung squamous cell carcinoma (LUSC), ovarian serous cystadenocarcinoma (OV), uterine corpus endometrial carcinoma (UCEC).

The WSI–genomic analysis comprised 2741 TCGA and 580 CPTAC patients across seven tumour types—BRCA, HNSC, KIRC, LUAD, LUSC, OV and UCEC—with analysis-specific sample sizes determined by the availability of both patient-level image predictions and deconstructSigs exposures (Figure 1). The TCGA prediction cohorts included 530 BRCA, 368 HNSC, 258 KIRC, 406 LUAD, 418 LUSC, 339 OV and 422 UCEC cases. The corresponding CPTAC cohorts comprised 77, 104, 83, 86, 103, 45 and 82 cases, respectively. Each tumour type contributed patient-level predictions for all 30 SBS targets, allowing the same signature landscape to be compared between the TCGA and CPTAC cohorts.

Each TCGA patient received an out-of-fold prediction generated by models trained without that patient. The corresponding TCGA-trained models were then applied to CPTAC without cohort-specific refitting.

### 3.2. Associations of Histology-Derived SBS Scores with Observed Exposures

**Figure 2:**
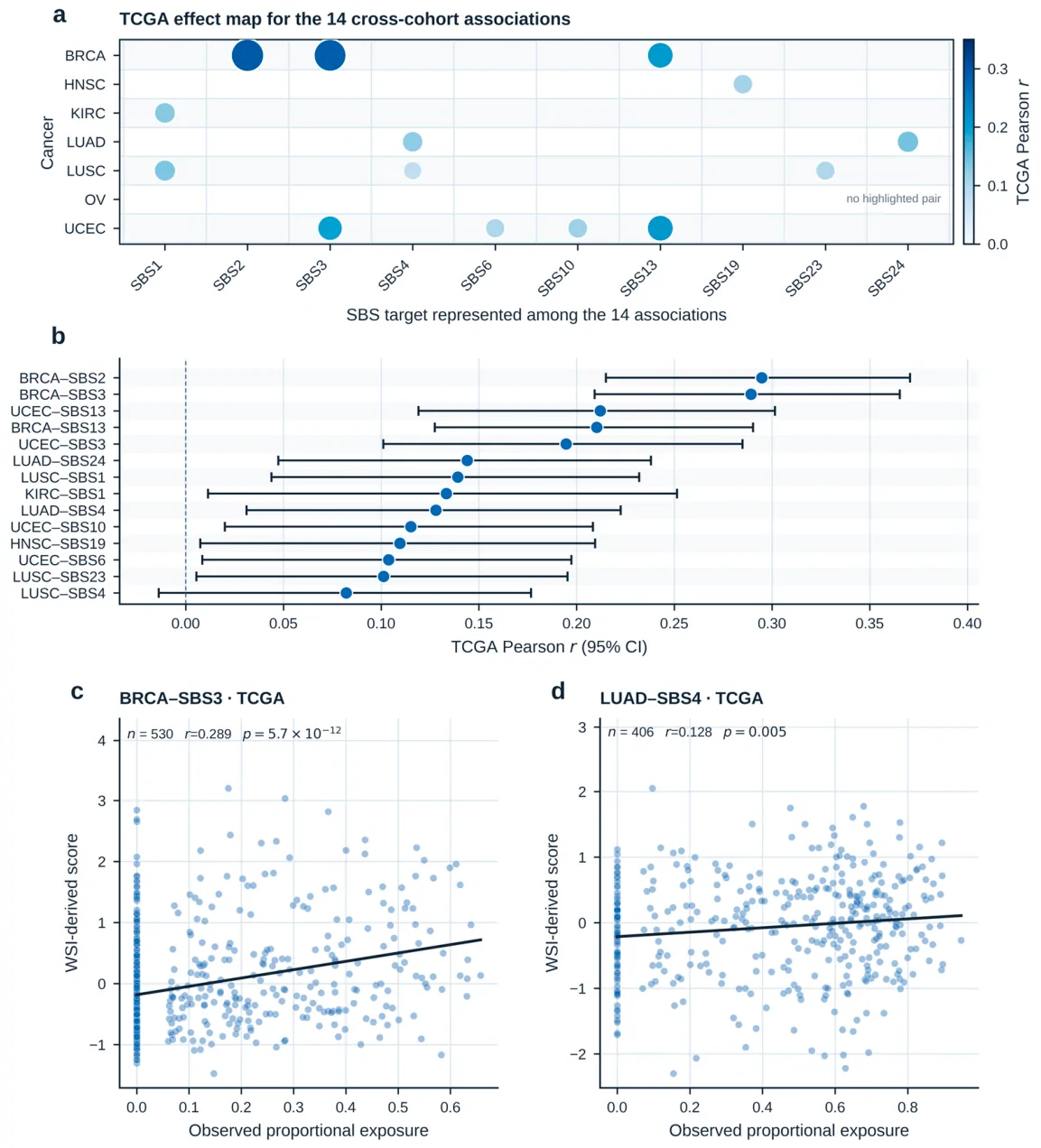
TCGA associations in the 210-pair SBS analysis. (**a**) Bubble map of the 14 nominally recurring cancer–SBS pairs that showed positive-direction *p* < 0.05 in both TCGA and CPTAC. Bubble area and blue intensity encode the TCGA Pearson r. OV is retained as an empty row because no OV pair met the cross-cohort criterion. The complete 210-pair TCGA landscape is shown in Supplementary Figure S1. (**b**) TCGA effects with Fisher-z 95% confidence intervals for the same 14 pairs. (**c**) BRCA–SBS3 (*n* = 530, r = 0.289, *p* = 5.68 × 10^−12^). (**d**) LUAD–SBS4 (*n* = 406, r = 0.128, *p* = 0.00492). Blue points in panels c and d represent individual TCGA patients; solid lines show least-squares fits. The dashed vertical line in panel b marks r = 0.

**Figure 3:**
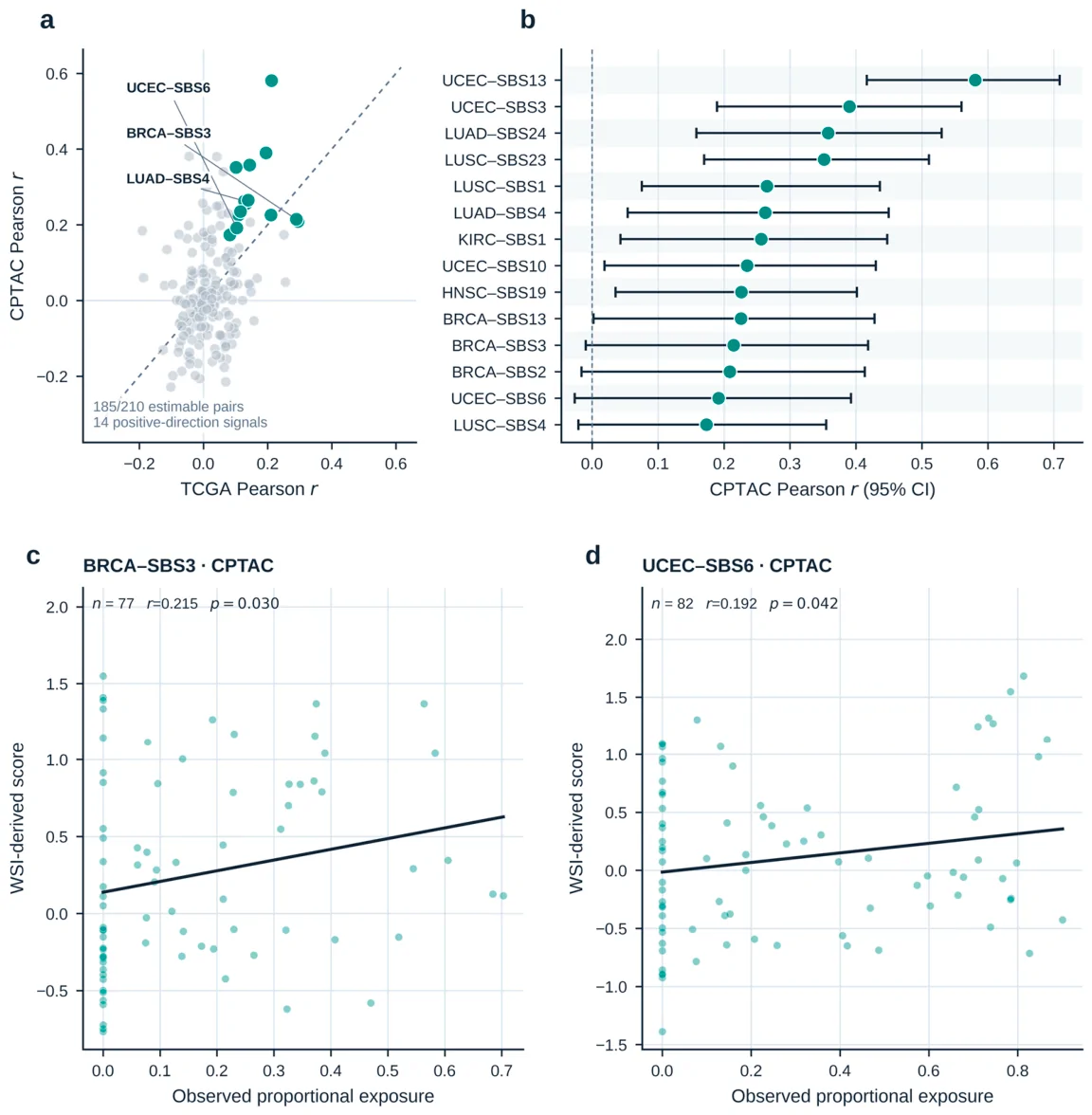
External validation of the SBS landscape in CPTAC. (**a**) TCGA and CPTAC Pearson effects for the 185 cancer–SBS pairs with correlations available in both cohorts. Teal symbols identify the 14 nominally recurring pairs that showed positive-direction *p* < 0.05 in both cohorts; grey symbols denote the remaining estimable pairs. The dashed diagonal marks equal correlations in the two cohorts. (**b**) CPTAC effects and Fisher-z 95% confidence intervals for these 14 pairs; the dashed vertical line marks r = 0. (**c**) BRCA–SBS3 (*n* = 77, r = 0.215, *p* = 0.0305). (**d**) UCEC–SBS6 (*n* = 82, r = 0.192, *p* = 0.0422). Teal points in panels c and d represent individual CPTAC patients; solid lines show least-squares fits.

Across the 210 tumour type–SBS combinations, Pearson correlations could be calculated in both cohorts for 185 pairs (73 positive–positive, 46 negative–negative and 66 discordant). The 119 direction-concordant pairs are a descriptive total. Negative–negative associations are not counted as successful positive-direction predictions. Thirty-five TCGA pairs and 34 CPTAC pairs had nominal positive-direction *p* < 0.05. Fourteen met this criterion in both cohorts, namely, BRCA–SBS2, BRCA–SBS3, BRCA–SBS13, HNSC–SBS19, KIRC–SBS1, LUAD–SBS4, LUAD–SBS24, LUSC–SBS1, LUSC–SBS4, LUSC–SBS23, UCEC–SBS3, UCEC–SBS6, UCEC–SBS10 and UCEC–SBS13 (Figure 2 and Figure 3). At an exploratory FDR threshold of 0.20, nine of these 14 associations remained significant in both cohorts. Complete two-cohort effects and confidence intervals are provided in Figure S1, with raw and adjusted *p*-values in Supplementary Data S3.

Several of these nominally recurring pairs corresponded to mutational processes with well-established aetiologies. HRD-associated BRCA–SBS3 showed positive correlations in both cohorts (TCGA r = 0.289, CPTAC r = 0.215). Smoking-associated SBS4 showed positive correlations in both cohorts for lung cancers, including LUAD (TCGA r = 0.128, CPTAC r = 0.263) and LUSC. APOBEC-associated signatures were represented by BRCA–SBS2, BRCA–SBS13 and UCEC–SBS13, while MSI-associated SBS6 was nominally positive in both UCEC cohorts (TCGA r = 0.104, CPTAC r = 0.192). Positive associations in both cohorts occurred across several mutational processes and tumour types, with generally modest effect sizes.

The Pearson findings were also examined using Spearman rank correlations. Across pairs with available correlations, Pearson and Spearman effect estimates were closely aligned, with correlations between the two effect-size measures of 0.924 in TCGA and 0.907 in CPTAC (Supplementary Figure S3a,b). Sign agreement between Pearson and Spearman estimates was observed for 174 of 200 TCGA pairs and 166 of 187 CPTAC pairs.

Using the unchanged image-derived scores, Sigfit and COSMIC v2 SigProfilerAssignment each retained positive correlations for all 14 original pairs in TCGA and 13 of 14 in CPTAC. With COSMIC v3.5 and v3.6, 12 of 13 comparable original pairs remained positive in TCGA and nine of 12 in CPTAC. Across the full set of comparable pairs, each alternative produced both increases and decreases in correlation. These comparisons used matched patients within each source and did not retrain the image models (Supplementary Data S3, signature_comparison).

### 3.3. Image-Input Sensitivity

The complete 210-pair input assessment compares the same 570 CPTAC patients using tile features from 2221 all-input source slides versus 1509 tumour-labelled slides. Four BRCA and six OV patients lack an explicitly tumour-labelled input in the matched metadata and are excluded only from this comparison. The primary analysis retained all 580 patients. All 14 original nominal pairs remained positively correlated after tumour-labelled slide restriction. Seven correlations increased and seven decreased. Among these 14 pairs, nominal CPTAC *p* < 0.05 was observed for 12 using all inputs and 10 after restriction. BRCA–SBS3 changed from r = 0.211 to 0.267, LUAD–SBS4 from 0.263 to 0.266 and UCEC–SBS13 from 0.581 to 0.584. UCEC–SBS3 and LUSC–SBS4 weakened from 0.390 to 0.344 and 0.173 to 0.072, respectively. Figure S3 summarises these pairs. Supplementary Data S3 reports input counts and all 210 paired correlations with confidence intervals. Observed correlations were higher in the FFPE-only group for some associations and in the frozen/OCT-only group for others. Performance differences by normal-slide inclusion could not be assessed reliably because comparison groups were absent or small. Group-specific results and sample sizes are provided in Supplementary Data S3.

### 3.4. Exploratory Associations with Genomic Features

**Figure 4:**
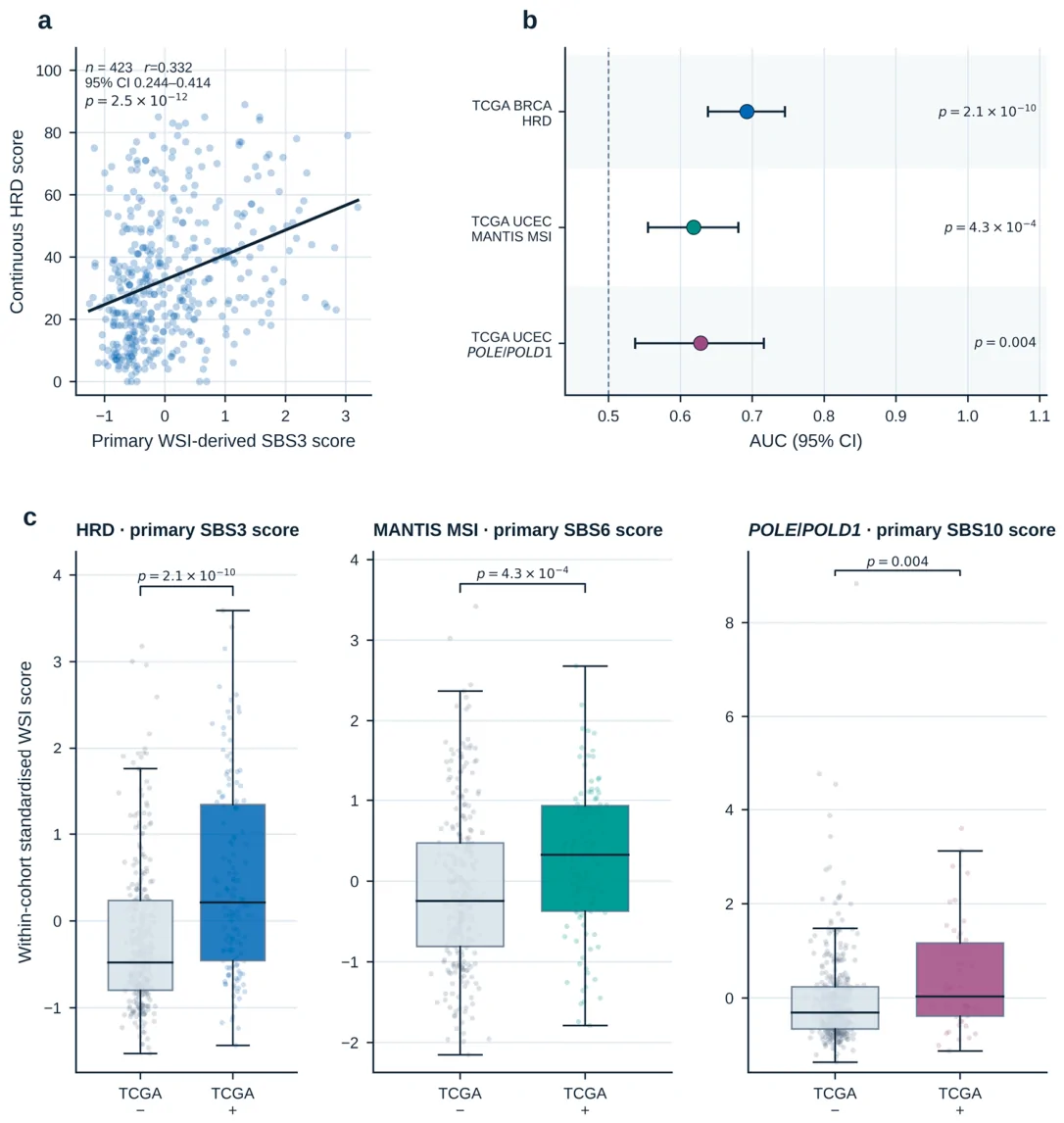
Exploratory associations of WSI-derived SBS scores with genomic features in TCGA. (**a**) SBS3 score versus continuous HRD (*n* = 423, r = 0.332, 95% CI 0.244–0.414, *p* = 2.47 × 10^−12^). (**b**) AUCs with bootstrap 95% confidence intervals for HRD-high using SBS3 (AUC = 0.693), MANTIS-defined MSI using the primary SBS6 score (AUC = 0.619) and the presence of the original qualifying *POLE* OR *POLD1* proofreading-domain substitutions using SBS10 (AUC = 0.628). The dashed vertical line in panel b marks AUC = 0.5. Blue, teal and purple denote HRD, MSI and the *POLE*/*POLD1* composite, respectively. (**c**) Score distributions for the same direct associations; grey indicates negative cases and the corresponding endpoint colour indicates positive cases. Scores in panel c were z-standardised within each clinical association cohort for display only. All association metrics were calculated from the out-of-fold scores. The *p*-value in panel a is from a two-sided Pearson correlation test. *p*-values for binary-group comparisons are from two-sided Mann–Whitney U tests. In panel c, centre lines indicate medians, boxes show the interquartile range (IQR), whiskers extend to the most extreme observations within 1.5 times the IQR, and points represent individual patients.

The exploratory genomic-feature analyses used primary TCGA out-of-fold SBS3, SBS6 and SBS10 scores. In TCGA BRCA, the WSI-predicted SBS3 score correlated with the continuous HRD score (*n* = 423, r = 0.332, 95% CI 0.244–0.414, *p* = 2.47 × 10^−12^, Figure 4). The same score discriminated HRD-high status, defined by an HRD score ≥ 42, with an AUC of 0.693 (95% CI 0.638–0.745, 132 positive patients among 423, *p* = 2.09 × 10^−10^).

In TCGA UCEC, the primary SBS6 score discriminated MANTIS-defined MSI status with an AUC of 0.619 (95% CI 0.555–0.681, 102 positive patients among 365, *p* = 4.34 × 10^−4^, Figure 4 and Supplementary Data S3).

The WSI-predicted SBS10 score similarly discriminated UCEC tumours carrying at least one of the originally listed qualifying *POLE* OR *POLD1* proofreading-domain substitutions with an AUC of 0.628 (95% CI 0.537–0.716, 47 positive patients among 422, *p* = 0.00409, Figure 4 and Supplementary Data S2). The mutation-positive group contained 44 *POLE*-positive and four *POLD1*-positive patients, including one patient positive for both genes. Double-positive patients were counted once, giving 47 qualifying-variant-positive and 375 qualifying-list-negative patients. No *POLD1*-specific performance inference was made.

Using the published MANTIS 0.4 threshold in TCGA increased the evaluated set from 365 to 409 of 422 UCEC patients and positive labels from 102 to 146. Thirteen patients lacked a continuous MANTIS score. Using the same SBS6 scores, the AUC was 0.592 (95% CI 0.536–0.649), compared with 0.619 under the original intermediate-score exclusion. In CPTAC UCEC, the primary SBS6 score yielded AUC = 0.573 (95% CI 0.429–0.709, 25 MSI-H patients among 82, two-sided *p* = 0.295). The same *POLE* OR *POLD1* list and rule identified six positives and 76 negatives. Five qualifying positive patients carried *POLE* p.P286R, and one carried *POLE* p.V411L. No qualifying *POLD1*-positive case was identified. The unchanged SBS10 score yielded AUC = 0.680 (95% CI 0.474–0.862, *p* = 0.150). The AUC estimates refer to the prespecified composite endpoint. *POLD1*-specific performance could not be evaluated. These endpoint analyses were exploratory (Figure S4 and Supplementary Data S3). External evaluation of the original BRCA genomic-scar HRD endpoint was not completed because equivalent labels were not obtained from the sources examined.

## 4. Discussion

This study evaluated histology-based inference of continuous SBS exposures across seven cancer types. The final ensemble outputs are standardised scores associated with genomic exposures, rather than calibrated exposure proportions. Fourteen cancer–signature pairs showed positive correlations with nominal *p* < 0.05 in both patient-disjoint TCGA evaluation and independent CPTAC patients, spanning six cancer types and several established mutational processes. Their recurrence in two patient populations supports a relationship between histology and these genomic exposures. Requiring nominal significance in both cohorts does not itself control the FDR across the screened associations. The cohort-specific adjusted *p*-values account for the multiple comparisons in this exploratory screen.

Previous computational pathology studies have established associations between H&E morphology and individual driver mutations, MSI, molecular subtypes and broad multi-omic biomarkers in single-cancer and pan-cancer settings [4,5,6,7,8,34,35]. Studies targeting HRD or MSI have further demonstrated attention-MIL regression or classification approaches and external validation for these clinically relevant phenotypes [9,10,11,12].

Valieris et al. used SBS3 and SBS20 activities to define HRD and MMR-deficient labels for WSI classification [13]. The Fuji multimodal pathology foundation model included binary mutational-signature inference during molecularly supervised pretraining [14]. Here, individual SBS exposures were regression targets in a fixed seven-cancer by 30-signature analysis, and the resulting models were assessed in tumour-type-matched CPTAC cohorts without refitting. The cross-cohort candidates include signatures related to homologous recombination deficiency (SBS3) [2], tobacco mutagenesis (SBS4), APOBEC activity (SBS2/SBS13) and mismatch repair deficiency (SBS6) [1,3]. These associations suggest links between mutational history and tissue appearance, although their cellular basis remains to be determined.

Our whole-tissue approach follows that of El Nahhas et al. [12], who used TCGA and CPTAC slides without prior tumour region segmentation. Valieris et al. [13] used background-filtered WSI tiles and subsequently identified tumour and non-tumour tissue among selected regions. All 14 original associations retained positive correlations after removing normal-labelled and unspecified slide inputs. These results indicate that normal-labelled or unspecified source inputs can influence individual correlations but do not fully account for the observed positive associations.

The genomic-feature analyses examined whether scores trained on SBS exposures retained information about related genomic states. The SBS3 association with HRD supports this interpretation in TCGA. In CPTAC, the SBS10 AUC was 0.680 for the predefined *POLE*/*POLD1* composite, while the SBS6 MSI AUC was 0.573. These external estimates had wide confidence intervals, particularly for the six polymerase-positive patients, whose qualifying variants were all in *POLE*. The analyses therefore remain exploratory and do not support *POLD1*-specific conclusions. Compatible genomic-scar HRD labels were unavailable for the matched CPTAC BRCA patients.

The input sensitivity analysis could not isolate the effect of normal tissue because normal-labelled and unspecified slides were excluded together. Slide labels do not identify tumour regions within individual tiles. Specimen-type comparisons used existing all-slide scores and were limited by small groups and incomplete annotations. The influence of normal-slide inclusion could not be assessed reliably because comparison groups were absent or small. Acquisition and site-specific histological characteristics can also affect molecular prediction [36]. Expert pathological review by pathologists with expertise across multiple tumour types is warranted to strengthen the biological interpretation of the attention examples. Such review could not be arranged, so the tissue types and possible artefacts in these examples remain unverified.

deconstructSigs was evaluated using simulated signature mixtures and TCGA data [22]. Although individual associations changed with alternative fitting approaches and reference catalogues, no alternative produced a consistent improvement across the evaluated cancer–signature pairs. We therefore retained the original deconstructSigs-based SBS1–SBS30 framework for consistency across cancer types and cohorts. Exome mutation counts, similarity between signatures, sparse exposures and the 0.06 contribution cutoff can affect the estimated targets.

## 5. Conclusions

Cancer-specific deep learning models inferred relative mutational signature activity from histology, with selected score–exposure associations observed in both TCGA and CPTAC. The complete seven-cancer, 30-signature analysis; adjustment for multiple testing; and input sensitivity analyses describe the strength and uncertainty of these associations. Further comparisons of signature estimates, expert pathological review and assessment in additional independent cohorts are needed to establish their biological and clinical relevance.

## Data Availability

TCGA data are available through the NCI Genomic Data Commons (https://portal.gdc.cancer.gov/). CPTAC images are available through The Cancer Imaging Archive (https://www.cancerimagingarchive.net/) and mutation data through doi:10.5281/zenodo.8394329. Derived results and source identifiers are supplied in Supplementary Data S1–S3. Controlled-access source data remain subject to repository requirements. TCGA clinical data were obtained from the Pan-Cancer Clinical Data Resource [37], and slide annotations were interpreted using the published pathology description [38]. The analysis code, software versions and environment specifications, preprocessing details, fold assignments, manifests and permissible derived results have been uploaded to a public GitHub repository (https://github.com/byeongchangsim/cancers-sbs (accessed on 22 October 2026); code snapshot: commit 4a6149661259).

## Supplementary Materials

The following supporting information can be downloaded at: https://www.mdpi.com/article/10.3390/cancers18213446/s1, Figure S1: Signature associations across cancer types; Figure S2: Target distributions, sparsity and catalogue counts; Figure S3: Robustness of score–exposure associations; Figure S4: Exploratory genomic-feature associations and MANTIS sensitivity; Figure S5: Paired TCGA/CPTAC attention examples. Supplementary Data S1 provides model settings, cohort characteristics and selection details. Supplementary Data S2 provides endpoint definitions, qualifying variants and the TCGA clinical scores and results used in Figure 4. Supplementary Data S3 provides complete association statistics, input sensitivity, clinical endpoint comparisons, target diagnostics, slide composition, specimen strata and alternative estimation comparisons. References [18,19,20,21,26,37,38] are also cited in the supplementary materials.
