# Supplementary material for: Deep Learning-Based Inference of Mutational Signature Exposures from Histology Across Tumour Types
Source: Cancers (Basel). 2026 Oct 25;18(21):3446. doi: 10.3390/cancers18213446 (PMC13761824; doi:10.3390/cancers18213446)
Supplement: Supplementary file 1 [file cancers-18-03446-s001.zip › Supplementary_Figures.pdf]

Figure S1. Signature associations across cancer types

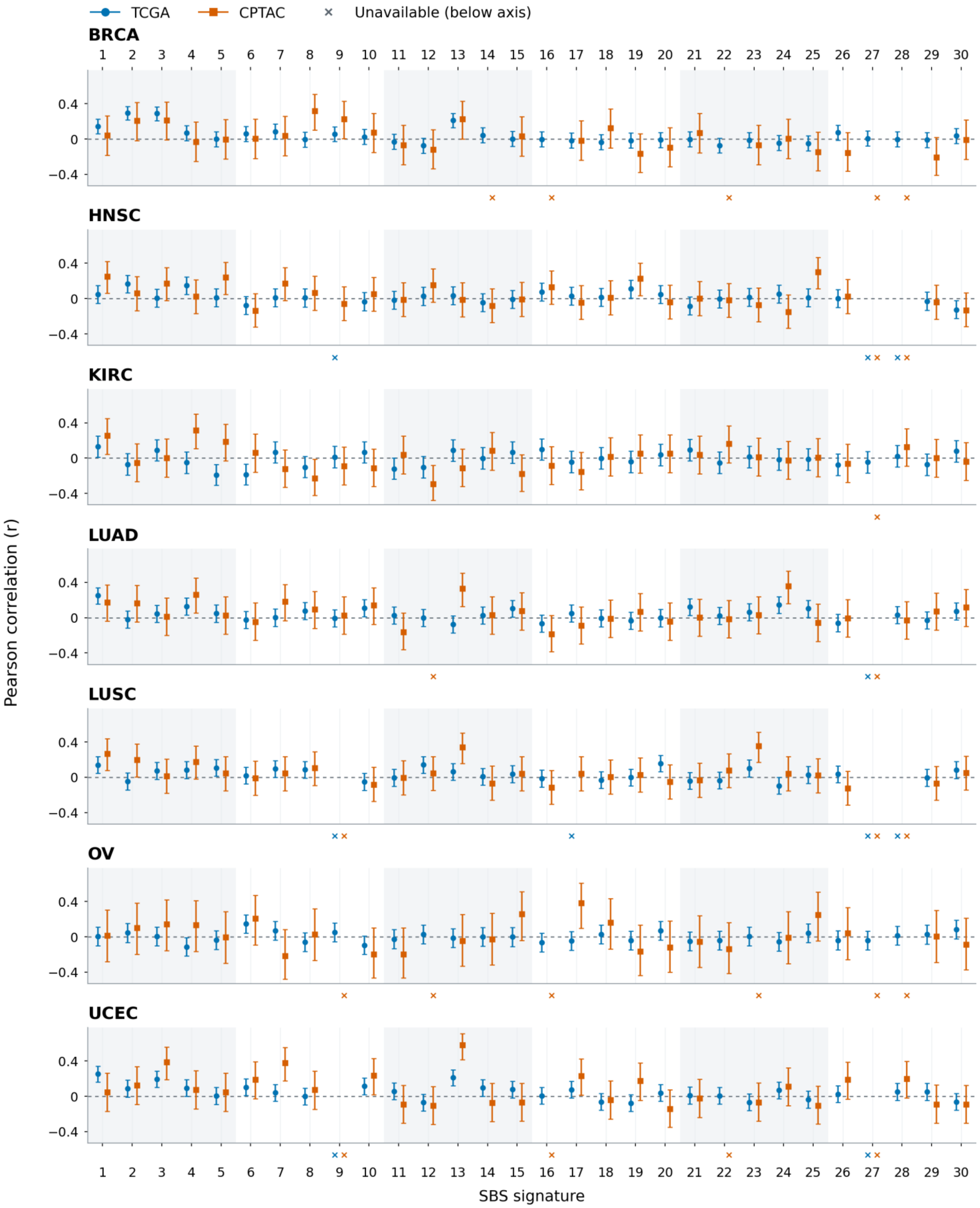

Pearson correlations between genomic SBS exposures and standardised image-derived scores are shown for all 210 cancer–signature combinations. Blue circles denote TCGA and orange squares denote CPTAC. Vertical bars show patient-level Fisher-z 95% confidence intervals. All panels use the same scale. Dashed lines mark r = 0. Crosses below the axes indicate unavailable correlations due to constant exposure or score values. Exact estimates, positive-direction one-sided P values and BH-adjusted P values across 210 tests separately in each cohort are provided in Supplementary Data S3 (paired_metrics).

Figure S2. Target distributions, sparsity and catalogue counts

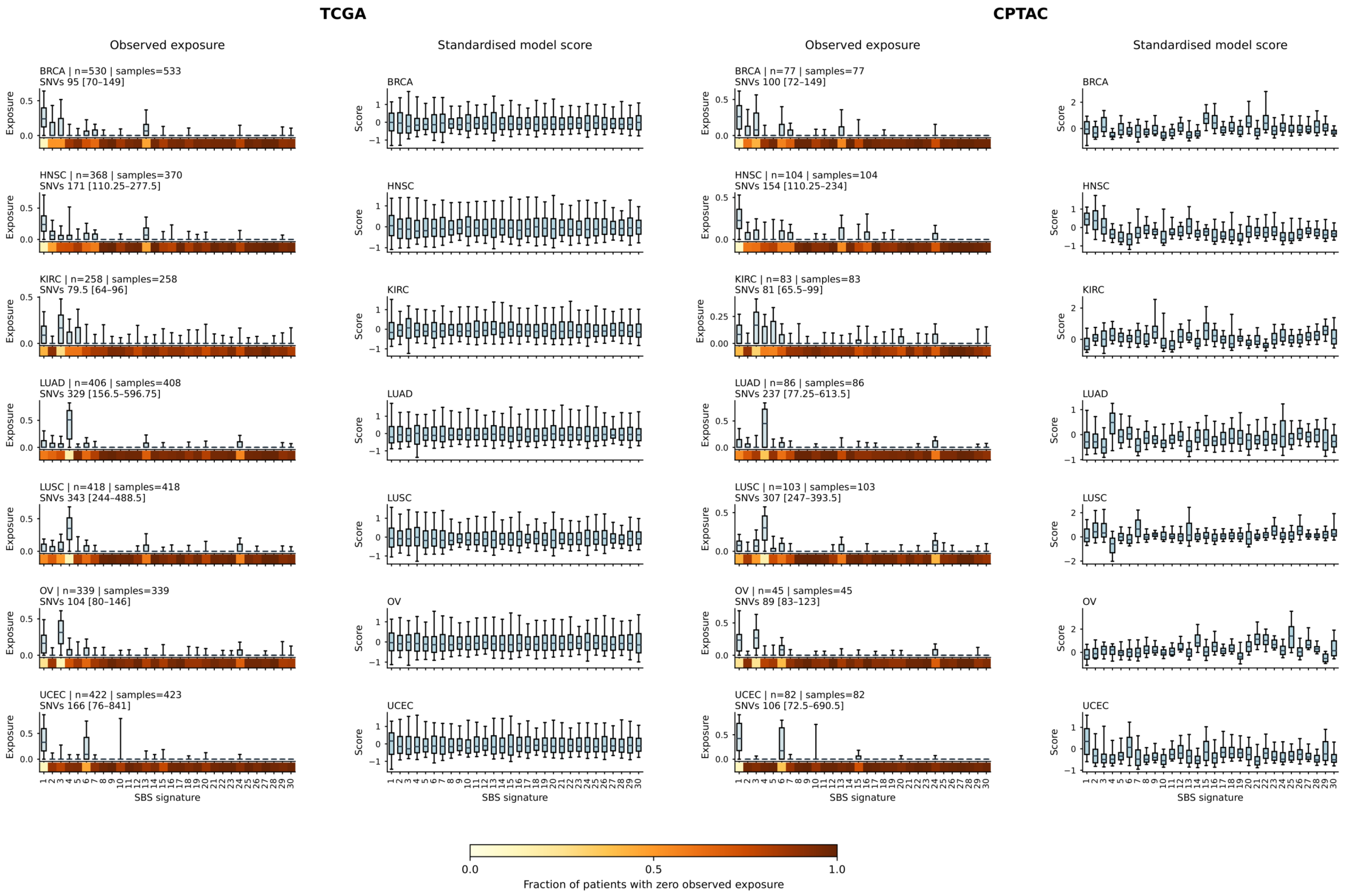

Observed SBS1–SBS30 exposures and standardised model scores are shown for TCGA (left two columns) and CPTAC (right two columns), with one cancer type per row. Boxes span the interquartile range, centre lines mark medians and whiskers extend to the 5th and 95th percentiles. Outliers are omitted. Yellow-to-brown strips show the fraction of patients with zero exposure for each signature. n denotes patients. Samples denotes included eligible genomic samples. Single-nucleotide variant (SNV) counts are sample-level medians [interquartile ranges]. Complete distributions, non-zero counts, mutation counts and eligibility summaries are provided in Supplementary Data S3 (distributions and mutation_summary).

Figure S3. Robustness of score–exposure associations

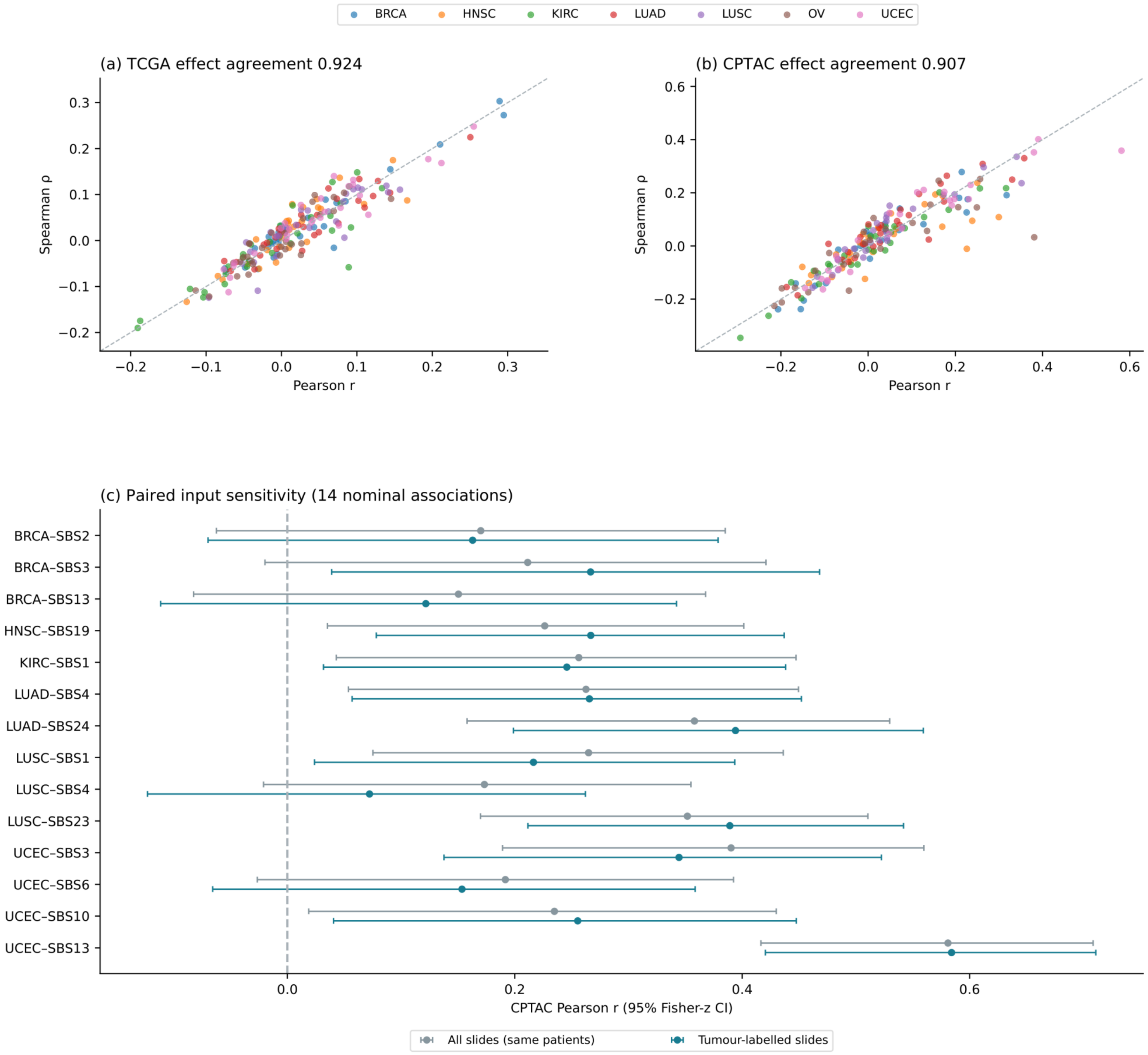

(a–b) Pearson r versus Spearman ρ in TCGA and CPTAC. Each point represents one cancer–signature pair, coloured by cancer type. Dashed diagonals mark equality. Effect agreement is the Pearson correlation between these two sets of coefficients across pairs. (c) The original 14 nominally replicated pairs under all-slide inputs (grey) and tumour-labelled-slide inputs (teal), evaluated in the same 570 CPTAC patients. Points show Pearson r and bars show Fisher-z 95% confidence intervals. The dashed line marks zero. Restriction uses source-slide annotations, not tile-level tumour classification. Ten patients lacking tumour-labelled inputs were excluded only from this comparison. The primary analysis retains 580 patients. Complete 210-pair sensitivity results and input counts are provided in Supplementary Data S3.

Figure S4. Exploratory genomic-feature associations and MANTIS sensitivity

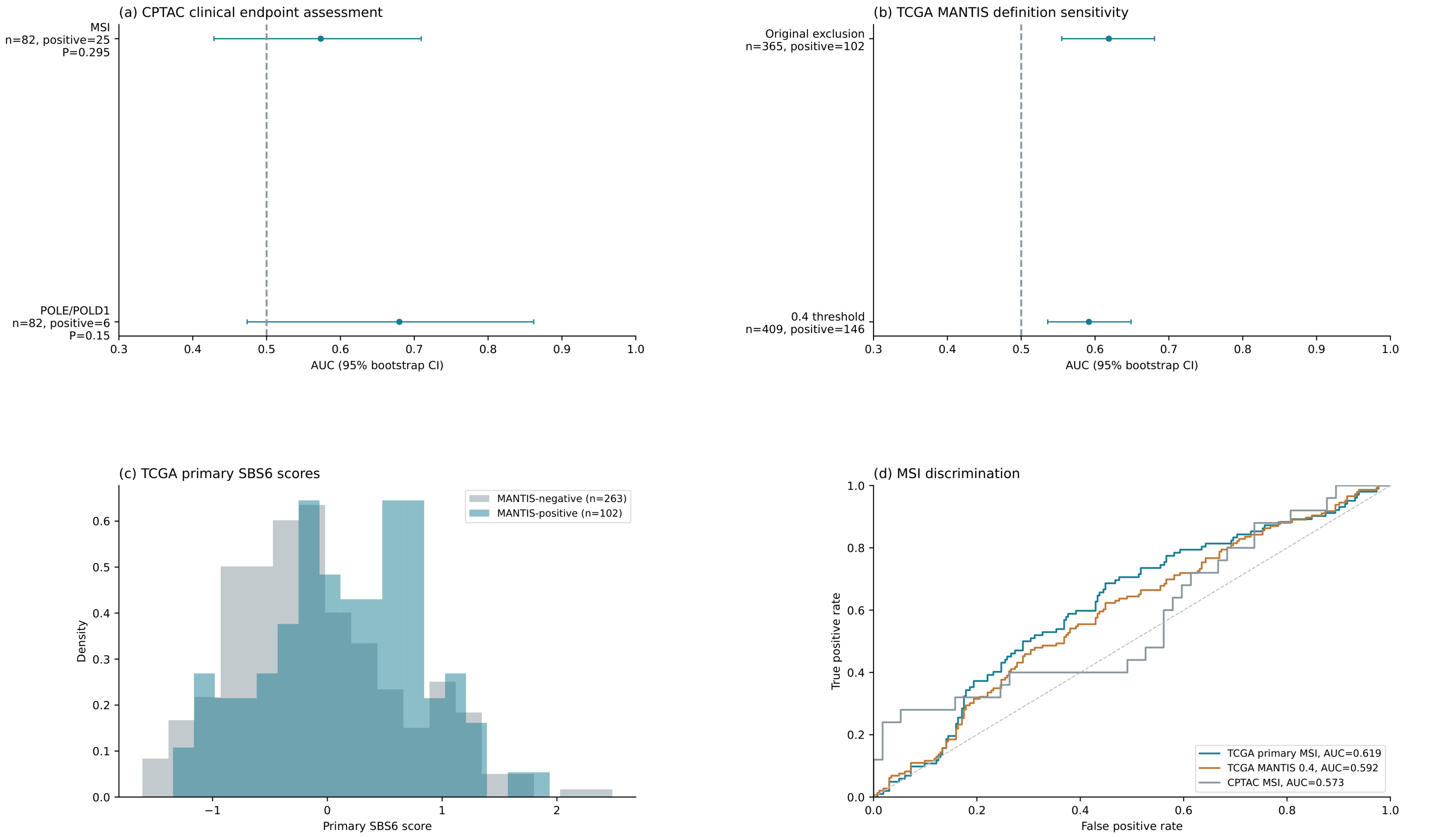

(a) External discrimination in 82 CPTAC UCEC patients using the primary SBS6 score for microsatellite instability (MSI, 25 positive cases) and the SBS10 score for the predefined POLE/POLD1 composite (six positive cases). P values are unadjusted, two-sided Mann–Whitney U tests. (b) TCGA MSI discrimination using the same SBS6 scores under the primary MANTIS rule (<0.4 negative, >0.6 positive, intermediate values excluded) and a 0.4 threshold (≤0.4 negative, >0.4 positive). In a–b, points show areas under the receiver operating characteristic curve (AUCs), bars show 95% confidence intervals from 10,000 class-stratified patient-bootstrap replicates and dashed lines mark AUC=0.5. (c) TCGA score distributions under the primary rule, with negatives in grey and positives in teal. (d) Receiver operating characteristic curves for the primary TCGA rule (teal), the TCGA 0.4 threshold (ochre) and CPTAC MSI labels (grey). The diagonal marks chance discrimination. The same primary SBS6 score is used in the signature analysis and MSI assessments. MSI labels were used only for evaluation. Endpoint definitions and results are provided in Supplementary Data S2 and S3.

Figure S5. Paired TCGA/CPTAC attention examples — UCEC–SBS13

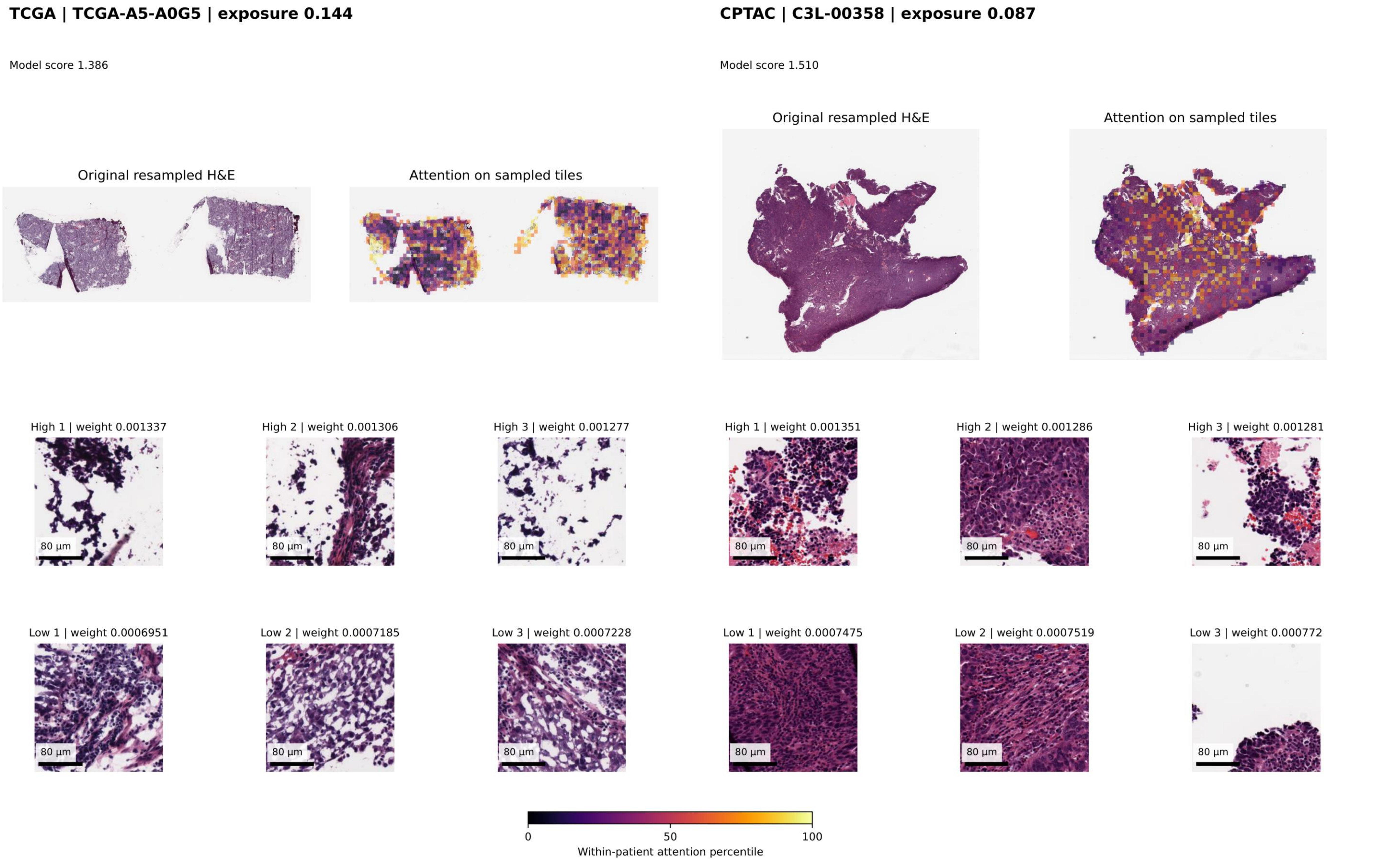

Eight illustrative cases cover UCEC–SBS13, UCEC–SBS3, BRCA–SBS3 and LUAD–SBS4, with TCGA on the left and CPTAC on the right. Cases were selected near the median positive genomic exposure among patients with tumour-labelled slides. Each case shows its exposure, standardised ensemble score, H&E overview, attention overlay and three higher- and three lower-weight tiles. Colours indicate within-patient attention percentile in one MIL component, from dark for lower weights to yellow for higher weights. Uncoloured regions were not sampled. Tile labels show component attention weights. Each crop spans 256 × 256 µm, with an 80 µm scale bar. These examples locate regions for inspection but do not establish tissue identity, morphological mechanisms or contributions to the complete ensemble. Expert pathological review was not performed.

Figure S5. Paired TCGA/CPTAC attention examples — UCEC–SBS3

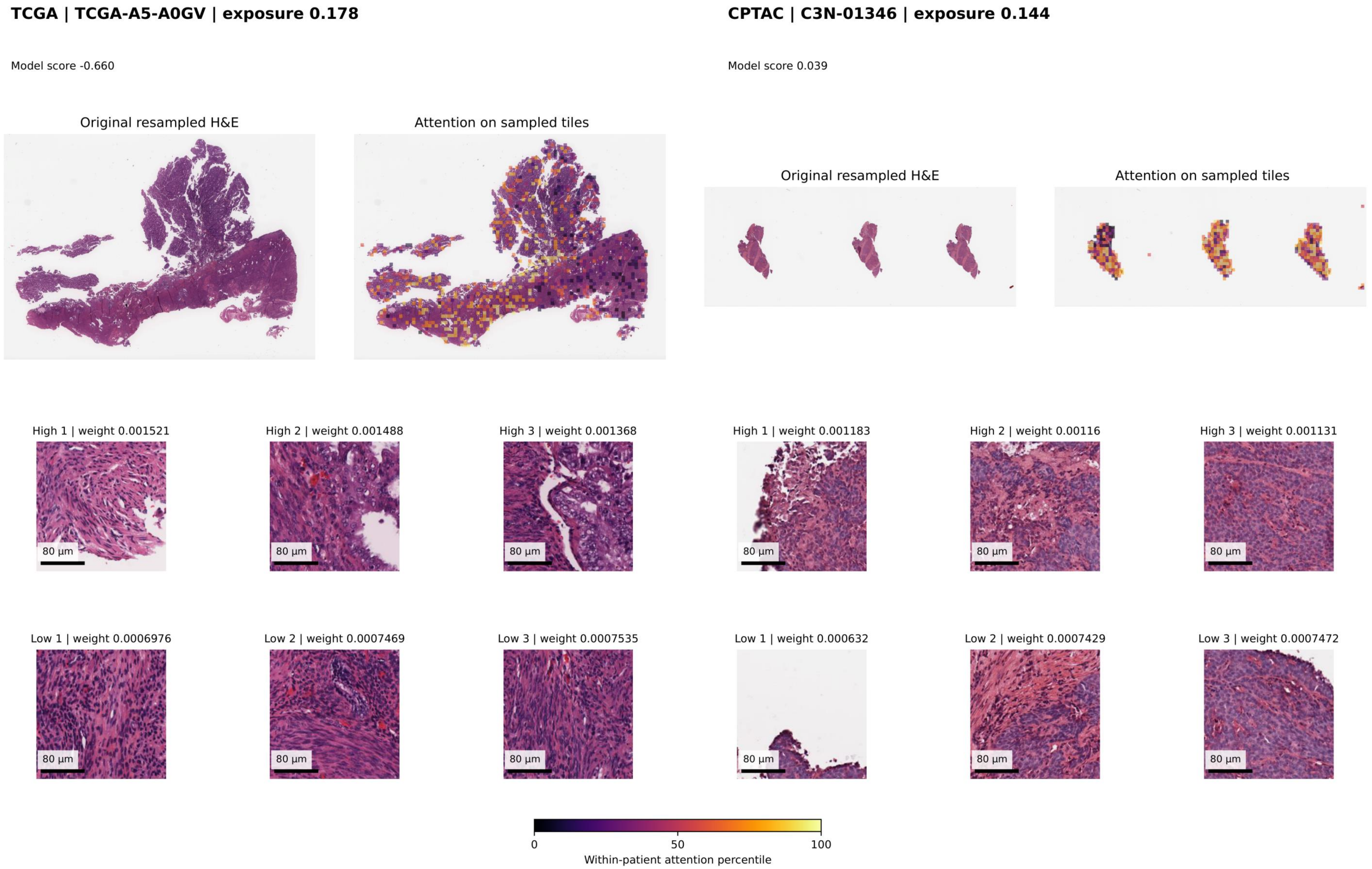

UCEC–SBS3 examples from TCGA (TCGA-A5-A0GV, left) and CPTAC (C3N-01346, right). Display conventions and case-selection criteria are described on the first page of Figure S5.

Figure S5. Paired TCGA/CPTAC attention examples — BRCA–SBS3

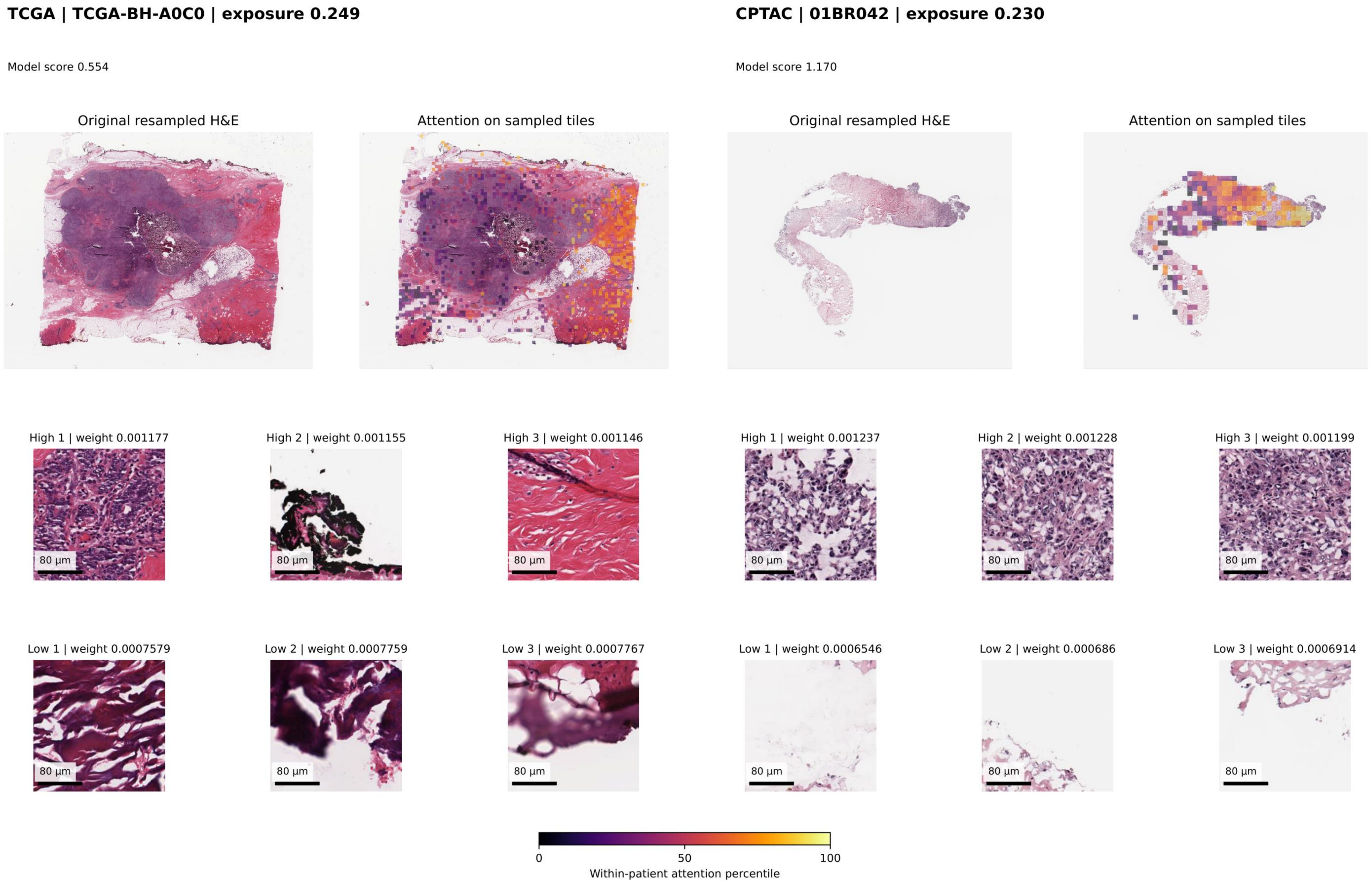

BRCA–SBS3 examples from TCGA (TCGA-BH-A0C0, left) and CPTAC (01BR042, right). Display conventions and case-selection criteria are described on the first page of Figure S5.

Figure S5. Paired TCGA/CPTAC attention examples — LUAD–SBS4

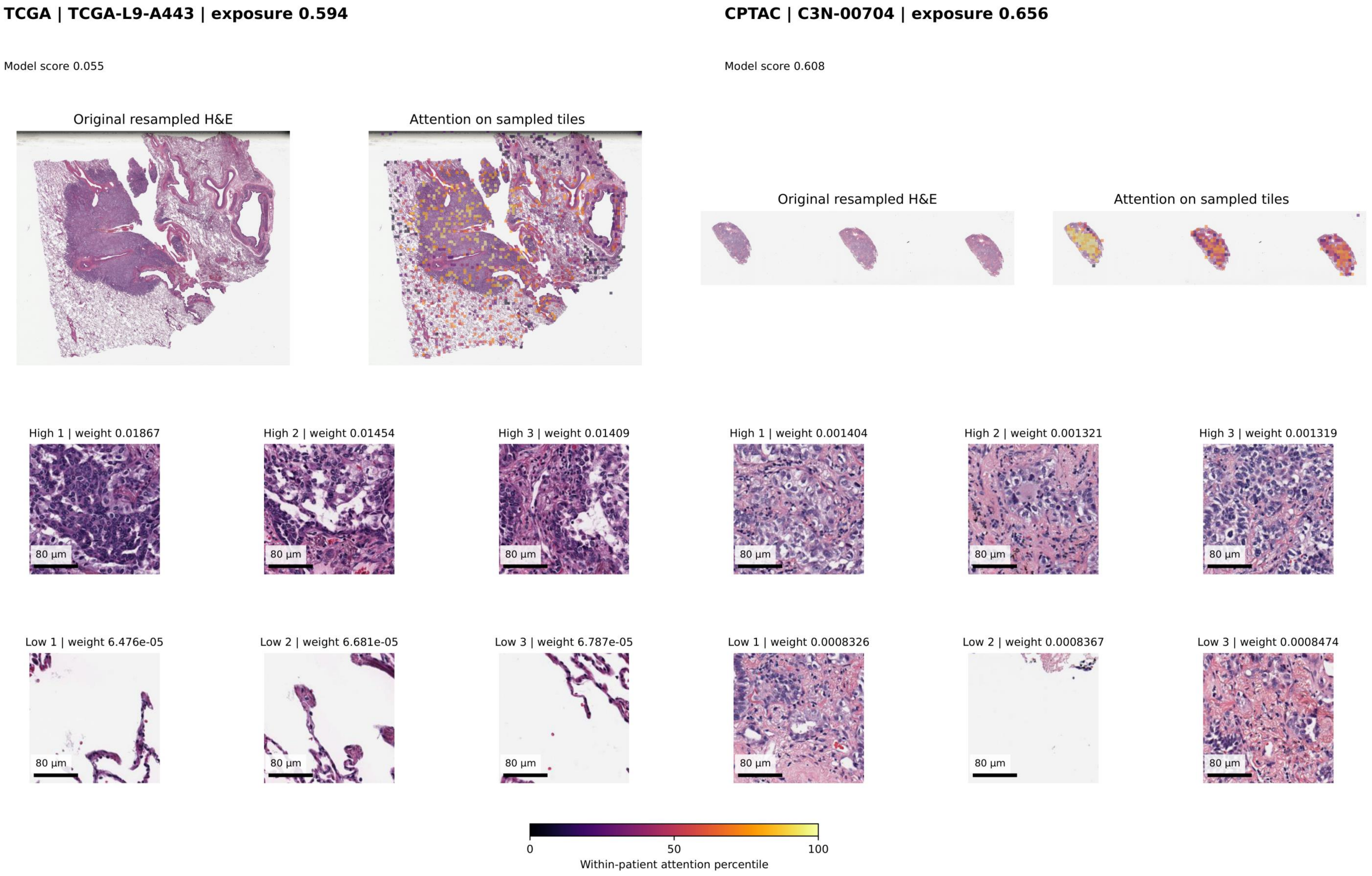

LUAD–SBS4 examples from TCGA (TCGA-L9-A443, left) and CPTAC (C3N-00704, right). Display conventions and case-selection criteria are described on the first page of Figure S5.
